# 2,5-Bis[(*E*)-2-phenyl­ethen­yl]-3,6-bis­(pyridin-2-yl)pyrazine

**DOI:** 10.1107/S2414314620003727

**Published:** 2020-03-17

**Authors:** Heiner Detert, Matthias Jochem, Dieter Schollmeyer

**Affiliations:** a Johannes Gutenberg University Mainz, Department of Chemistry, Duesbergweg 10-14, 55099 Mainz, Germany; Goethe-Universität Frankfurt, Germany

**Keywords:** crystal structure, heterocycles, conjugated oligomers

## Abstract

The mol­ecule of the title compound, C_30_H_22_N_4_, exhibits inversion symmetry adopting the shape of a St Andrew’s Cross. It shows dihedral angles between adjacent aryl units of around 50° whereas torsion angles of *ca* 10° are found along the aryl­ene vinyl­ene path.

## Abstract 2

The title mol­ecule has inversion symmetry, adopting the shape of a St Andrew’s cross. It shows dihedral angles between adjacent aryl units of around 50° whereas torsion angles of *ca* 10° are found along the aryl­ene vinyl­ene path.

## Graphical abstract

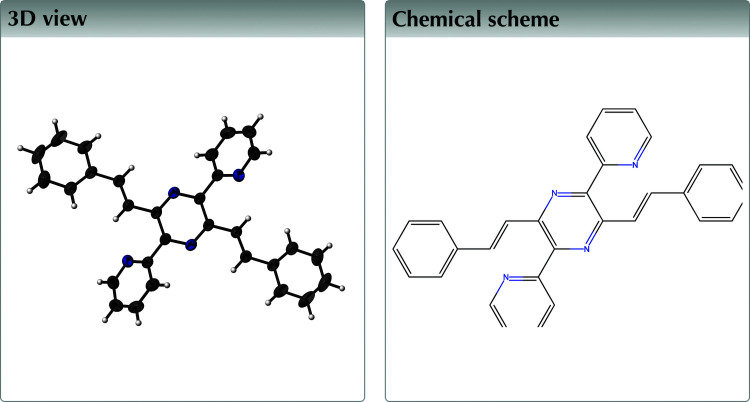

## Structure description

The title compound 2,5-(*E*,*E*)-distyryl-3,6-di-(2-pyrid­yl)pyrazine, C_30_H_22_N_4_, was prepared as a reference chromophore in a project on pyrazine-centered materials, solvatochromic dyes (Schmitt *et al.*, 2008 ▸, Wink & Detert, 2013 ▸) and liquid crystals (Röder *et al.*, 2019 ▸; Schmitt *et al.*, 2011 ▸).

**Figure 1:**
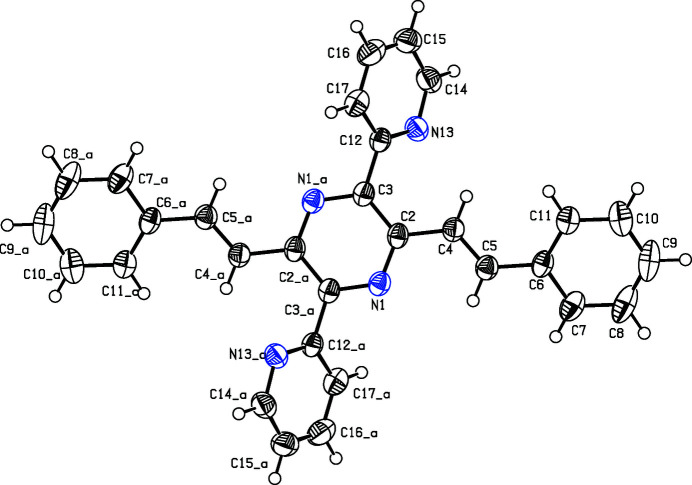
Perspective view of the title compound. Displacement ellipsoids are drawn at the 50% probability level. The second part of the mol­ecule is generated by the symmetry operation 1 − *x*, 1 − *y*, 1 − *z*.

**Figure 2:**
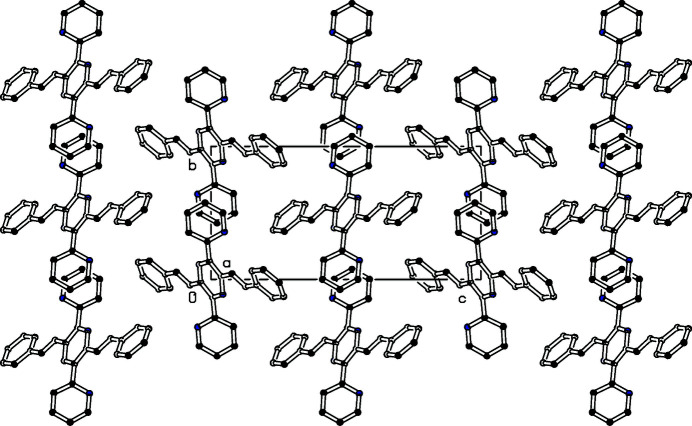
Partial packing diagram of the title compound. View along the *a* axis.

The mol­ecule has the shape of a centrosymmetrical St Andrew’s cross (Fig. 1 ▸). The central pyrazine ring as well as the vinyl­ene groups and the peripheral pyridine and phenyl rings are totally planar. A dihedral angle of 48.07 (6)° at the teraryl axis is nearly identical to those in a related compound with phenyl rings (50.8, 48.6°, Schmitt *et al.*, 2013 ▸). Torsion angles along the distyryl axis are −170.21 (15)°, (phenyl-vin­yl) and −169.56 (14)° (vinyl-pyrazine). The packing is shown in Fig. 2 ▸.

## Synthesis and crystallization

The title compound was prepared from 2,5-dimethyl-3,6-di(2-pyrid­yl)pyrazine (Kolb, 1896 ▸) (0.08 g) and benzaldehyde (0.13 g) in 35 ml of DMF by the action of 0.34 g potassium *t*-butyl­ate. The base was added in portions to the stirred and cooled (30 min at 273 K) solution. After 4 h at ambient temperature, the mixture was poured into water, extracted with ethyl acetate and the organic layers were washed, dried (Na_2_SO_4_) and concentrated. Purification by chromatography on solica gel with toluene/ethyl acetate (20/1) as eluent, *R*_f_ = 0.33. Yield: 40 mg, 30%.

^1^H NMR (CDCl_3_, 400 MHz): 8.83 (*dd*, *J* = 4.9 Hz, *J* = 1.5 Hz, 2 H), 8.23 (*d*, *J* = 7.8 Hz, 2 H), 8.03 (*s* = 2*d*, *J* = 16.1 Hz, 4 H), 7.95 (*dt*, *J* = 7.8 Hz, *J* = 1.9 Hz, 2 H), 7.58 (*d*, 4 H), 7.43 (*ddd*, *J* = 7.8 Hz, *J* = 4.9 Hz, *J* = 1.5 Hz, 2H), 7.36 (*t*, *J* = 7.3 Hz, 4 H), 7.29 (*dt*, *J* = 7.3 Hz, *J* = 1.4 Hz, 2H); ^13^C NMR (CDCl_3_, 100 MHz): 157.1, 148.9, 148.3, 145.9, 137.3, 137.0, 135.2, 128.7, 127.6, 125.4, 124.7, 123.6; IR (ATR): 3009, 2988, 2926, 2853, 2688, 1473, 1448, 1276, 1254, 1135, 1086, 1040, 962, 901, 89, 752, 699, 621; MS (APCI): calculated for C_30_H_22_N_4_+H^+^): 439.1917, found 439.1908.

## Refinement

Crystal data, data collection and structure refinement details are summarized in Table 1 ▸.

## Supplementary Material

Crystal structure: contains datablock(s) I, global. DOI: 10.1107/S2414314620003727/bt4090sup1.cif

Structure factors: contains datablock(s) I. DOI: 10.1107/S2414314620003727/bt4090Isup2.hkl

Supporting information file. DOI: 10.1107/S2414314620003727/bt4090Isup3.cml

CCDC reference: 1989990

Additional supporting information:  crystallographic information; 3D view; checkCIF report

## Figures and Tables

**Table 1:** Experimental details

Crystal data	
Chemical formula	C_30_H_22_N_4_
*M*_r_	438.51
Crystal system, space group	Monoclinic, *P*2_1_/*c*
Temperature (K)	193
*a*, *b*, *c* (Å)	7.0953 (8), 8.9310 (8), 18.219 (2)
β (°)	95.490 (9)
*V* (Å^3^)	1149.2 (2)
*Z*	2
Radiation type	Mo *K*α
μ (mm^−1^)	0.08
Crystal size (mm)	0.53 × 0.32 × 0.06

Data collection	
Diffractometer	STOE *IPDS* 2T
Absorption correction	–
No. of measured, independent and observed [*I* > 2σ(*I*)] reflections	5878, 2718, 1622
*R*_int_	0.029
(sin θ/λ)_max_ (Å^−1^)	0.659

Refinement	
*R*[*F* ^2^ > 2σ(*F* ^2^)], *wR*(*F* ^2^), *S*	0.043, 0.110, 0.99
No. of reflections	2718
No. of parameters	154
H-atom treatment	H-atom parameters constrained
Δρ_max_, Δρ_min_ (e Å^−3^)	0.17, −0.14

Computer programs: *X-AREA* and *X-RED* (Stoe & Cie, 1996 ▸), *SIR2004* (Burla *et al.*, 2005 ▸), *SHELXL2018* (Sheldrick, 2015 ▸) and *PLATON* (Spek, 2020 ▸).

## full crystallographic data

### Crystal data

**Table:**

C_30_H_22_N_4_	*F*(000) = 460
*M**_r_* = 438.51	*D*_x_ = 1.267 Mg m^−^^3^
Monoclinic, *P*2_1_/*c*	Mo *K*α radiation, λ = 0.71073 Å
*a* = 7.0953 (8) Å	Cell parameters from 3402 reflections
*b* = 8.9310 (8) Å	θ = 2.5–28.2°
*c* = 18.219 (2) Å	µ = 0.08 mm^−^^1^
β = 95.490 (9)°	*T* = 193 K
*V* = 1149.2 (2) Å^3^	Plate, yellow
*Z* = 2	0.53 × 0.32 × 0.06 mm

### Data collection

**Table:**

STOE IPDS 2T diffractometer	1622 reflections with *I* > 2σ(*I*)
Radiation source: sealed X-ray tube, 12 x 0.4 mm long-fine focus	*R*_int_ = 0.029
Detector resolution: 6.67 pixels mm^-1^	θ_max_ = 27.9°, θ_min_ = 2.5°
rotation method scans	*h* = −9→9
5878 measured reflections	*k* = −10→11
2718 independent reflections	*l* = −23→21

### Refinement

**Table:**

Refinement on *F*^2^	Primary atom site location: structure-invariant direct methods
Least-squares matrix: full	Hydrogen site location: inferred from neighbouring sites
*R*[*F*^2^ > 2σ(*F*^2^)] = 0.043	H-atom parameters constrained
*wR*(*F*^2^) = 0.110	*w* = 1/[σ^2^(*F*_o_^2^) + (0.0551*P*)^2^] where *P* = (*F*_o_^2^ + 2*F*_c_^2^)/3
*S* = 0.99	(Δ/σ)_max_ < 0.001
2718 reflections	Δρ_max_ = 0.17 e Å^−^^3^
154 parameters	Δρ_min_ = −0.14 e Å^−^^3^
0 restraints	

### Special details

**Table:**

Geometry. All esds (except the esd in the dihedral angle between two l.s. planes) are estimated using the full covariance matrix. The cell esds are taken into account individually in the estimation of esds in distances, angles and torsion angles; correlations between esds in cell parameters are only used when they are defined by crystal symmetry. An approximate (isotropic) treatment of cell esds is used for estimating esds involving l.s. planes.
Refinement. Hydrogen atoms attached to carbons were placed at calculated positions and were refined in the riding-model approximation with C–H = 0.95 Å, and with *U*_iso_(H) = 1.2 *U*_eq_(C).

### Fractional atomic coordinates and isotropic or equivalent isotropic displacement parameters (Å^2^)

**Table:**

	*x*	*y*	*z*	*U*_iso_*/*U*_eq_	
N1	0.56710 (15)	0.38847 (13)	0.45640 (6)	0.0371 (3)	
C2	0.65303 (18)	0.52229 (16)	0.46177 (7)	0.0356 (3)	
C3	0.58305 (19)	0.63537 (16)	0.50618 (7)	0.0349 (3)	
C4	0.82169 (19)	0.54154 (17)	0.42232 (8)	0.0377 (3)	
H4	0.898305	0.627307	0.433494	0.045*	
C5	0.8747 (2)	0.44665 (17)	0.37180 (8)	0.0394 (3)	
H5	0.796135	0.361702	0.361337	0.047*	
C6	1.04162 (19)	0.45956 (17)	0.33065 (8)	0.0385 (4)	
C7	1.0601 (2)	0.3624 (2)	0.27172 (8)	0.0524 (4)	
H7	0.965446	0.288996	0.259487	0.063*	
C8	1.2135 (3)	0.3716 (2)	0.23111 (10)	0.0691 (6)	
H8	1.223164	0.305063	0.190953	0.083*	
C9	1.3524 (3)	0.4758 (3)	0.24812 (10)	0.0697 (6)	
H9	1.458129	0.481619	0.219953	0.084*	
C10	1.3377 (2)	0.5724 (2)	0.30648 (10)	0.0585 (5)	
H10	1.433770	0.644817	0.318544	0.070*	
C11	1.1840 (2)	0.56400 (19)	0.34735 (8)	0.0444 (4)	
H11	1.175609	0.630726	0.387515	0.053*	
C12	0.66436 (18)	0.78758 (17)	0.51362 (8)	0.0364 (3)	
N13	0.69586 (16)	0.85842 (14)	0.45085 (7)	0.0415 (3)	
C14	0.7551 (2)	1.00017 (19)	0.45675 (10)	0.0487 (4)	
H14	0.774730	1.052537	0.412735	0.058*	
C15	0.7892 (2)	1.0746 (2)	0.52227 (11)	0.0545 (5)	
H15	0.831478	1.175580	0.523436	0.065*	
C16	0.7610 (2)	1.0003 (2)	0.58648 (10)	0.0555 (5)	
H16	0.786532	1.048282	0.632899	0.067*	
C17	0.6949 (2)	0.85434 (19)	0.58234 (9)	0.0467 (4)	
H17	0.671004	0.801209	0.625662	0.056*	

### Atomic displacement parameters (Å^2^)

**Table:**

	*U*^11^	*U*^22^	*U*^33^	*U*^12^	*U*^13^	*U*^23^
N1	0.0403 (6)	0.0387 (7)	0.0329 (6)	0.0050 (5)	0.0070 (5)	0.0027 (5)
C2	0.0356 (7)	0.0408 (9)	0.0310 (7)	0.0055 (6)	0.0066 (6)	0.0059 (6)
C3	0.0365 (7)	0.0376 (8)	0.0312 (7)	0.0064 (6)	0.0056 (5)	0.0055 (6)
C4	0.0389 (7)	0.0382 (8)	0.0371 (7)	0.0031 (6)	0.0087 (6)	0.0026 (7)
C5	0.0414 (7)	0.0412 (8)	0.0362 (8)	0.0023 (7)	0.0069 (6)	0.0029 (7)
C6	0.0382 (7)	0.0472 (9)	0.0306 (7)	0.0095 (6)	0.0054 (5)	0.0028 (7)
C7	0.0546 (10)	0.0671 (12)	0.0359 (8)	0.0094 (8)	0.0072 (7)	−0.0088 (8)
C8	0.0652 (12)	0.1046 (17)	0.0397 (9)	0.0236 (12)	0.0156 (8)	−0.0111 (10)
C9	0.0503 (10)	0.1129 (18)	0.0494 (10)	0.0206 (12)	0.0227 (8)	0.0132 (12)
C10	0.0414 (8)	0.0791 (13)	0.0562 (10)	0.0031 (9)	0.0107 (7)	0.0166 (10)
C11	0.0429 (8)	0.0512 (10)	0.0400 (8)	0.0060 (7)	0.0079 (6)	0.0034 (7)
C12	0.0312 (7)	0.0392 (8)	0.0391 (8)	0.0059 (6)	0.0057 (6)	0.0000 (7)
N13	0.0395 (7)	0.0396 (7)	0.0462 (7)	−0.0004 (6)	0.0073 (5)	0.0044 (6)
C14	0.0401 (8)	0.0426 (10)	0.0644 (11)	0.0005 (7)	0.0093 (7)	0.0066 (8)
C15	0.0393 (8)	0.0422 (10)	0.0820 (13)	0.0020 (7)	0.0065 (8)	−0.0087 (10)
C16	0.0446 (9)	0.0606 (11)	0.0602 (11)	0.0061 (8)	0.0001 (8)	−0.0226 (9)
C17	0.0437 (8)	0.0532 (10)	0.0433 (9)	0.0070 (7)	0.0049 (6)	−0.0042 (8)

### Geometric parameters (Å, º)

**Table:**

N1—C3^i^	1.3356 (17)	C9—C10	1.381 (3)
N1—C2	1.3410 (18)	C9—H9	0.9500
C2—C3	1.4136 (19)	C10—C11	1.380 (2)
C2—C4	1.4638 (19)	C10—H10	0.9500
C3—C12	1.478 (2)	C11—H11	0.9500
C4—C5	1.332 (2)	C12—N13	1.3442 (18)
C4—H4	0.9500	C12—C17	1.385 (2)
C5—C6	1.4655 (19)	N13—C14	1.335 (2)
C5—H5	0.9500	C14—C15	1.368 (2)
C6—C11	1.387 (2)	C14—H14	0.9500
C6—C7	1.396 (2)	C15—C16	1.376 (3)
C7—C8	1.376 (2)	C15—H15	0.9500
C7—H7	0.9500	C16—C17	1.385 (2)
C8—C9	1.369 (3)	C16—H16	0.9500
C8—H8	0.9500	C17—H17	0.9500
			
C3^i^—N1—C2	118.95 (12)	C10—C9—H9	120.2
N1—C2—C3	119.76 (12)	C11—C10—C9	120.21 (17)
N1—C2—C4	117.14 (13)	C11—C10—H10	119.9
C3—C2—C4	123.06 (13)	C9—C10—H10	119.9
N1^i^—C3—C2	121.29 (13)	C10—C11—C6	120.97 (16)
N1^i^—C3—C12	115.04 (12)	C10—C11—H11	119.5
C2—C3—C12	123.65 (12)	C6—C11—H11	119.5
C5—C4—C2	124.26 (14)	N13—C12—C17	122.83 (15)
C5—C4—H4	117.9	N13—C12—C3	116.76 (13)
C2—C4—H4	117.9	C17—C12—C3	120.31 (14)
C4—C5—C6	126.81 (14)	C14—N13—C12	117.03 (14)
C4—C5—H5	116.6	N13—C14—C15	124.01 (16)
C6—C5—H5	116.6	N13—C14—H14	118.0
C11—C6—C7	117.79 (14)	C15—C14—H14	118.0
C11—C6—C5	123.22 (13)	C14—C15—C16	118.66 (17)
C7—C6—C5	118.99 (14)	C14—C15—H15	120.7
C8—C7—C6	120.94 (17)	C16—C15—H15	120.7
C8—C7—H7	119.5	C15—C16—C17	118.92 (16)
C6—C7—H7	119.5	C15—C16—H16	120.5
C9—C8—C7	120.52 (17)	C17—C16—H16	120.5
C9—C8—H8	119.7	C16—C17—C12	118.51 (16)
C7—C8—H8	119.7	C16—C17—H17	120.7
C8—C9—C10	119.56 (16)	C12—C17—H17	120.7
C8—C9—H9	120.2		
			
C3^i^—N1—C2—C3	−0.4 (2)	C9—C10—C11—C6	−0.2 (2)
C3^i^—N1—C2—C4	177.52 (12)	C7—C6—C11—C10	0.7 (2)
N1—C2—C3—N1^i^	0.4 (2)	C5—C6—C11—C10	−179.48 (14)
C4—C2—C3—N1^i^	−177.38 (12)	N1^i^—C3—C12—N13	−130.40 (13)
N1—C2—C3—C12	−177.97 (12)	C2—C3—C12—N13	48.11 (17)
C4—C2—C3—C12	4.2 (2)	N1^i^—C3—C12—C17	46.03 (17)
N1—C2—C4—C5	12.6 (2)	C2—C3—C12—C17	−135.47 (14)
C3—C2—C4—C5	−169.56 (13)	C17—C12—N13—C14	−1.5 (2)
C2—C4—C5—C6	−179.88 (13)	C3—C12—N13—C14	174.83 (12)
C4—C5—C6—C11	9.9 (2)	C12—N13—C14—C15	1.6 (2)
C4—C5—C6—C7	−170.21 (15)	N13—C14—C15—C16	−0.1 (2)
C11—C6—C7—C8	−0.8 (2)	C14—C15—C16—C17	−1.6 (2)
C5—C6—C7—C8	179.34 (15)	C15—C16—C17—C12	1.7 (2)
C6—C7—C8—C9	0.5 (3)	N13—C12—C17—C16	−0.1 (2)
C7—C8—C9—C10	−0.1 (3)	C3—C12—C17—C16	−176.32 (13)
C8—C9—C10—C11	−0.1 (3)		

Symmetry code: (i) −*x*+1, −*y*+1, −*z*+1.
